# Re-evaluating evidence for adaptive mutation rate variation

**DOI:** 10.1038/s41586-023-06314-y

**Published:** 2023-07-26

**Authors:** Long Wang, Alexander T. Ho, Laurence D. Hurst, Sihai Yang

**Affiliations:** 1 State Key Laboratory of Pharmaceutical Biotechnology, School of Life Sciences, Nanjing University, Nanjing, China; 2 The Milner Centre for Evolution, Department of Biology and Biochemistry, University of Bath, Bath, UK

**Keywords:** Mutation, Molecular evolution

arising from J. G. Monroe et al. *Nature* 10.1038/s41586-021-04269-6 (2022)

Although mutation rates vary within genomes, suggestions^1,2^ that more selectively important DNA has a lower mutation rate are contentious not least because unbiased estimation of the mutation rate is challenging^3^. Monroe et al.^4^ (hereafter Monroe) also report that in *Arabidopsis* more important sequences have lower mutation rates and, while overlooking similar claims^1^, suggest that this challenges “a long-standing paradigm regarding the randomness of mutation”^4^. We find, however, that their mutation calling has abundant sequencing and analysis artefacts explaining why their data are not congruent with well-evidenced mutational profiles. As the key trends associated with sequence importance are consistent with well-described mutation-calling artefacts and are not resilient to reanalysis using the higher-quality components of their data, we conclude that their claims are not robustly substantiated.

In principle, identifying new mutations is simple: one sequences genomes of close relatives and identifies new differences between them. There are, however, multiple pitfalls. For example, incorrect mapping of short reads to the genome can result in erroneous, and commonly clustered, mutation calls. Further, as the rate of sequencing errors is orders of magnitude higher than the rate of mutation, these errors must be excluded. Robust rules for mutation calling, such as requiring multiple independent sequence reads from both strands supporting the same mutation, can obviate many issues.

We expect higher than normal error rates in Monroe as, to identify somatic mutations, they relaxed their previous^5^ stringency in mutation calling (Supplementary Methods). To assay the impact of this, we compared their mutation calls to those generated by a conventional pipe. We find only 3.7% (*n* = 160) concordance with Monroe’s 4,322 filtered putative mutations, 61% of which are uncallable. We term the 96.3% of Monroe’s mutations that fail conventional filters as low quality (LQ). Their previous data^5^ using a more stringent pipe (we dub this Weng data) agree with our analysis: 94.2% agreement vis-à-vis mutations ‘confidently’ called, only 1.4% uncallable. Prima facie, most of Monroe’s mutation calls thus may well be unsafe. This is supported by analysis of the profile of LQ mutations as this is different to the higher-quality calls: they are of a qualitatively different type (intergenic, intronic and so on) compared with high-quality datasets (that mutually agree; Extended Data Fig. 1a,b) and have a different mononucleotide mutational profile (Extended Data Fig. 1c).

Deviations of this magnitude are unlikely to be accounted for by a somatic versus germline difference. Instead, Monroe’s data are different largely because they are enriched for sequencing and analysis artefacts. We consider two artefact fingerprints. First, in Illumina sequencing^6,7^, a base can be erroneously replaced by a bleeding one within^8,9^ (for example, AAAGAAA appears as AAAAAAA) or in the vicinity of^6,7^ (for example, AAAAC appears as AAAAA) homopolymeric runs. Second, failure to eliminate poor-quality reads and mapping artefacts will overreport clustering of putative mutations.

More than half (54%) in the LQ data are bleeding-type putative mutations within 5 base pairs of A/T homopolymers (Extended Data Fig. 1d), compared with 24% in the high-quality (HQ) Weng set. Within genic regions (exonic + intronic), the proportion is 67.5% in the LQ data, 91% of which are intronic. Depending on the protocol, the error is biased to bleed-over of either AT or GC residues, but not both^6^. It is then notable that the bias is particular to A/T homomeric runs with 1.5% in HQ Weng and 1.2% in LQ near GC runs (Extended Data Fig. 1d). This AT bias artefact is reported for related Illumina machines^6^.

Of the 2,247 bleed mutations near homopolymeric runs, 1,149 (51.1%) are immediately next to or within the runs. Of the remaining 1,098, at least 648 cluster with other bleed errors (for example, AAAAACACACA is read as AAAAAAAAAAA giving three putative mutations). These bear the hallmarks of artefacts: typically only one strand is affected, all of the putative mutations are seen in the same read and their rate decays as a function of distance from the true end of the run. As also expected from the profile of sequencing errors^6^, the probability of a mutation being called increases with the length of the homopolymer: in introns, regression of log_10_(putative mutations per base pair of homopolymeric sequence) predicted by run length, slope = 0.27, Pearson’s *r*^2^ = 0.87, *P* = 0.0006, degrees of freedom (df) = 6.

For Monroe’s somatic mutations (recalled from Weng’s vcf data) unassociated with homomeric runs (46% of their mutations), most are clustered (2 mutations within 10 base pairs of each, 27.5% of all mutations) or unexpectedly common (>10 samples, 8.7% of all mutations), indicative of mis-mapping issues. Only 2.5% in the Weng HQ data are clustered. Many of Monroe’s putative mutations are associated with more than one error: about 34% are associated with A/T homomeric runs and in a tight cluster. As centromeres are prone to mapping errors^10^, mis-mapping probably explains why 40.9% of LQ mutations are centromeric (see, for example, Extended Data Fig. 1e) compared with 27.9% in Weng HQ.

We do not suppose these to be all of the errors. Whereas Monroe call 773,141 mutations using our sequence^11^, using the same HaplotypeCaller-GVCF calling method^12^, with default parameters and without any further filtering, we identify only 31,486 raw indels and 72,516 raw single nucleotide polymorphisms (all but 17 of which are unsafe). This gross excess of mutations in Monroe is due to an analysis error on their part (see correction from Monroe et al.^13^).

Analysis and sequencing errors explain many of the core claims of Monroe. They report that the mutation rate alters markedly around transcription start sites (TSSs) and stop sites (TTSs), arguing that this provides evidence that gene bodies are mutationally protected. We simulated random errors associated with A/T homopolymeric runs and derived a distribution that is a near-perfect match to their somatic data (Fig. 1a).

**Fig. 1:**
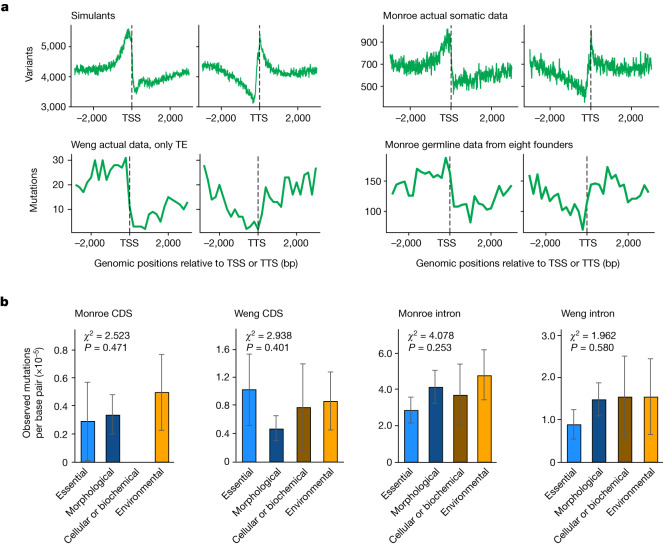
Core claims of Monroe et al. are error artefacts. **a**, Top row: the profile of errors (misascribed as mutations) expected around TSS and TTS attributable to the errors associated with A/T homopolymeric runs. We simulated 2 million errors associated with A/T homopolymeric runs and used the Monroe script to generate the left plot. The data are an exact match to their somatic data calls (reproduced here as the top right plot, figure reused from Monroe et al.^4^ under Creative Commons Attribution 4.0 International License). Bottom row: germline mutations in TEs (left panel). Observed mutations in germline unmasked. bp, base pair. **b**, Essential genes do not have a low mutation rate. The second claim of Monroe to substantiate that mutation is lower in more functionally important sequences is that essential genes have the lowest mutation rates (their Fig. 3c). To substantiate this, they seem to have used many thousands of unfiltered calls. We repeat the analysis using filtered data, either Weng or Monroe, including indels. In neither case is there significant heterogeneity (we provide *χ*^2^ values for all comparisons, df = 3, but for Monroe CDS numbers are so small that these calculations are not valid and presented for completeness alone). Tests are one-sided in the sense that we call significance only if there is heterogeneity not if they are more similar than expected. Tests are two-sided in the sense that we ask about deviation from null in any direction. Unification of the two datasets does not alter conclusions: CDS, *χ*^2^ = 3.3; intron, *χ*^2^ = 5.76, *P* > 0.05 for all without multi-test correction. Error bars are s.e.m. across gene samples for which sample sizes are: essential (*n* = 719), morphological (*n* = 861), cellular or biochemical (*n* = 297) and environmental (*n* = 522) for CDS analysis (Monroe CDS and Weng CDS), and essential (*n* = 671), morphological (*n* = 789), cellular or biochemical (*n* = 270) and environmental (*n* = 452) for the intron analysis (Monroe intron and Weng intron). For representation of the underlying data, see Extended Data Fig. 2.

Monroe also claimed a low mutation rate in essential genes as evidence for mutational protection for more important sequences (their Fig. 3c). However, to do this, they included orders of magnitude more putative mutations than in their filtered datasets. We repeated their analysis using the Weng data and the Monroe data (their filtering). In neither, nor in the merged dataset, is there heterogeneity in the mutation rate between gene classes in coding sequence (CDS) or intron (Fig. 1b and Extended Data Fig. 2). Indeed, in the best data (Weng), essential genes have the highest mutation rate per base pair of CDS (Fig. 1b and Extended Data Fig. 2).

Artefacts explain other heterogeneities in the mutation rate. Weng’s data report a plausible intron to CDS per base pair ratio of about 0.91:1 (paired *t*-test on normalized dinucleotide mutation rates, *P* = 0.58, df = 95), whereas Monroe’s data report an unprecedented 5.2 to 1 ratio (paired *t*, *P* = 3.5 × 10^−8^, df = 95). This comparison is especially informative as it controls for transcription-associated mutational effects. Much of this higher intronic rate in Monroe’s data is attributable to homopolymeric run artefacts as CDS has fewer, and less error prone, runs (Supplementary Results).

The artefacts are also evident in the profile of mutations called (Extended Data Fig. 1f). Counts of the 96 dinucleotide mutations from the Monroe and Weng data are discordant (*χ*^2^ = 1,516, *P* < 2 × 10^−16^, df = 95). The most common dinucleotide mutations in the Monroe data end AA or TT as the resolved mutational event, with G/C mutated to the neighbouring A/T being especially discrepant (Extended Data Fig. 1f). The mutational events terminating AA/TT contribute 34.4% of the relative normalized mutations in the Monroe set but only 21.6% in Weng’s.

The Monroe data also incorrectly predict the mutational equilibrium frequency of AA/TT dinucleotides compared to observed frequencies. Using the 16 × 16 normalized mutational matrix for the Monroe and the Weng data individually, we predict mutational equilibrium dinucleotide content^14^ and compare with intergenic dinucleotide content. The Weng data are not influenced by AA/TT calls (*P* = 0.38), whereas in the Monroe data AA/TT are over-called outliers (*P* = 0.003; Extended Data Fig. 1g).

This neighbour base matching affecting both A and T in Monroe’s data is an expected bleed artefact with no biological basis. By contrast, we expect CpG>TpG mutations to be common given well-described methylated CpG hyperinstability^15^. In Weng’s data, but much less so Monroe’s, this is the case (Extended Data Fig. 1f).

Although Monroe’s claim that the mutation rates are lower at more functionally important sites seems to be highly influenced by artefacts, nonetheless, the mutation rate is not uniform. In some part this is because transposable elements (TEs) have high mutation rates and TEs are rare in gene bodies. In *Arabidopsis*, cytosine methylation-mediated TE suppression^16^ should lead to C instability. In the Weng data, 65% of mutations in TE are CG, CHH or CHG versus 51% in intergenic non-TE (for example, 5.2:1 ratio of CpG>TpG per CG, TE to intergenic non-TE). In (robust) germline data there is a higher mutation rate in TEs than elsewhere, including the best comparator, non-TE intergenic sequence: TE versus non-TE intergenic sequence, mean ratio per dinucleotide = 3.93 (paired *t*-test on normalized dinucleotide rates, *P* < 3 × 10^−7^, df = 95). This TE enrichment largely explains why in germline data mutation rates are higher 5′ of TSS and 3′ of TTS, TEs being enriched outside transcribed domains (Fig. 1a).

Although TE mutational enrichment is seen in Monroe HQ data (Extended Data Fig. 1a,b), it is not seen in the Monroe data in toto (paired *t*-test on normalized dinucleotide rates, *P* = 0.9). Given this and the failure to capture well-described methyl C instability^15^, although we do not doubt that epigenetic marks such as methylation can affect mutation, the correlations evidenced by Monroe between various marks and mutation rate variation should be treated with the same caution as their claim that mutation is rarer in more important sequences.

## Reporting summary

Further information on research design is available in the Nature Portfolio Reporting Summary linked to this article.

## Online content

Any methods, additional references, Nature Portfolio reporting summaries, source data, extended data, supplementary information, acknowledgements, peer review information; details of author contributions and competing interests; and statements of data and code availability are available at 10.1038/s41586-023-06314-y.

## Supplementary information

Supplementary Information This file contains Supplementary Methods and Results.

Reporting Summary

## Figures and Tables

**Extended Data Fig. 1:**
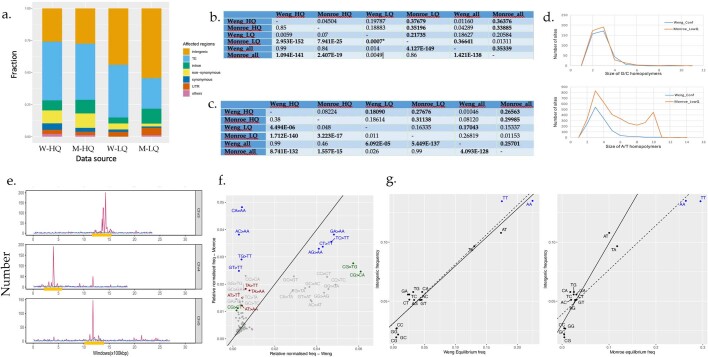
Mutational properties in different mutation data sets. We consider the data from Weng et al.^5^ and Monroe et al.^4^. We reanalysed both datasets from raw files and split the data into confident mutation calls (HQ) and low-quality calls (LQ). The samples sizes are 1743, 160, 107, 4162 in Weng high confident (W-HQ), Monroe high confident (M-HQ), Weng low quality (W-LQ), Monroe low quality (M-LQ). **a** is a visual representation of the frequency of each class of mutation, **b** are the Euclidean distances between the frequency vectors (upper section), including the two full data sets. The values below the diagonal are chi^2^ P values with v = 7, based on raw counts omitting any rows where both were zero (indicated *, v = 6). Those significant after Bonferonni correction are highlighted in bold. Tests are one sided in the sense that we call significance only if there is heterogeneity not if they are more similar than expected. Tests are two sided in the sense that we ask about deviation from null in any direction. **c**. Relative rates of different mononucleotide mutations. Above the diagonal, Euclidean distances between the 12 element vectors of relative mutation frequencies. Below the diagnonal, P from chi^2^ on raw counts (v = 11), with those significant after Bonferonni correction highlighted in bold. Tests are one sided in the sense that we call significance only if there is heterogeneity not if they are more similar than expected. Tests are two sided in the sense that we ask about deviation from null in any direction. **d**. rates of potential bleed errors in proximity to homopolymeric runs of different length (top panel A or T runs, bottom panel, G or C runs) for Weng HQ (i.e. Confident) and Monroe LQ calls. Y axis is number of homomeric runs with associated bleed type mutations. **e**. Distribution of mutations on chromosomes 3, 4 and 5. Centromere is shown as orange block. Weng HQ data is in blue, Monroe LQ data in red. **f**. Relative normalised dinucleotide mutation frequencies in the Weng et al. and Monroe et al. data. In each data set we determined the absolute number of each dinucleotide-associated mutation. We then determined the normalised rate by dividing observed rates by numbers of each dinucleotide in the genome, this providing a rate per bp. The sum rate for each set was calculated and the normalised rates divided by this sum to provide a relative normalised rate. The line of slope 1 indicates equivalence between the two data sets. In blue and red are all the dinucleotide based events that terminate either AA or TT after mutation. In blue are those mutating C/G residues, in red, A/T residues. CG starting dinucleotides are in green. For clarity most other data points are represented by dots alone. **g**. Predicted and observed dinucleotide frequencies. Observed dinucleotide frequencies are from intergenic sequence. Mutational equilibrium analytically derived as in ref. ^14^. Left panel, Weng et al. full data, right panel, Monroe et al. full data. To test for AA/TT concordance, we consider slopes from regression of observed and predicted, including (dashed) and omitting (solid) AA and TT. If AA and TT are unduly influential, we expect a significant difference in slopes. Difference in slopes was tested by *t* test with df = 26 (Monroe data, *t* = 3.39, *P* = 0.0028, Weng data, *t* = −0.26, *P* = 0.38). The test is two-sided.

**Extended Data Fig. 2:**
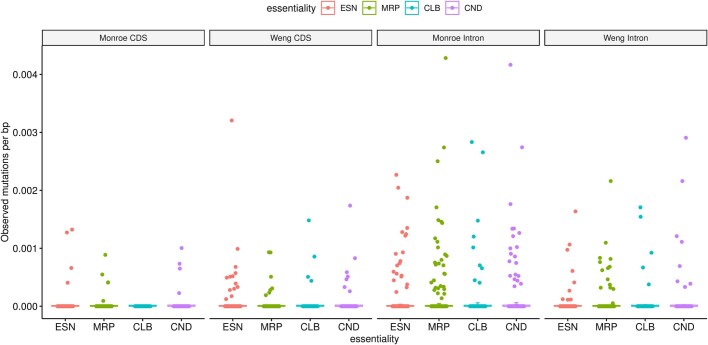
Representation of Fig. 1b showing underlying data points. Note that for most genes there are no mutations in the reduced data sets hence most data sits at y = 0.

## Data Availability

The data to replicate the analyses and figures in the paper and the Extended Data are available at https://github.com/wl13/reanalysis1.

## Code availability

The code to replicate the analyses and figures in the paper and the Extended Data is available at https://github.com/wl13/reanalysis1.
