# Supplementary material for: Re-evaluating evidence for adaptive mutation rate variation
Source: Nature. 2023 Jul 26;619(7971):E52–6. doi: 10.1038/s41586-023-06314-y (PMC10371861; doi:10.1038/s41586-023-06314-y)
Supplement: Supplementary file 1 — This file contains Supplementary Methods and Results. [file 41586_2023_6314_MOESM1_ESM.pdf]

nature portfolio

https://doi.org/10.1038/s41586-023-06314-y

Supplementary information

Re-evaluating evidence for adaptive mutation rate variation

In the format provided by the authors and unedited

Supplementary Methods

Our pipe for mutation determination

Monroe relied solely on a single lossy GVCF-produced vcf file and no read-level filtering (their Ext Data Fig. 1a). For calling somatic mutations, however, more stringent conditions, such as read-level filtering, are necessary. Contrary to Monroe, our protocol collects sufficient read-level metrics to determine whether a mutation (either germline or somatic) is reliable or not. We counted the supporting reads in forward and reverse strands using VarScan readcounts[4], and also checked their mapping quality, insert size, and paired orientations. Those with strand bias (read support in only one strand), poor mapping quality (mostly mapping quality = 0), and abnormal insert size or orientation are considered as low-quality or uncallable. We also compared across samples to see whether the so-called "mutated reads" are unique to <=10 samples (already a relaxed criterion as the MA lines are independent to each other so should not share somatic mutations). Those present in > 10 samples we assume to be repeated artefacts (e.g., mapping artefacts).

Supplementary results

Evidence that the intron-CDS discrepancy in rates is largely owing to sequencing artefacts

A/T homomeric runs (of any length) are rarer in CDS (11.9% of CDS) than introns (22.6%), and introns have proportionally more longer runs, the ratio of the sum span of intronic to CDS A/T runs also increasing in an exponential fashion with run length ($\log_{10}$(intron/CDS spans) ~ run length, slope = 0.27, r^2=0.97, P=6 x 10^{-6}, d.f. = 6). While with no masking of homopolymeric runs the intron to CDS mutation rate (per bp) ratio is 6.8 fold higher in Monroe's data than Weng data, in masked analysis this reduces to 1.99. We conclude that the much higher rate of "mutation" in intron than in CDS as reported by Monroe et al is attributable to a failure to filter out sequencing artefact.
